# Immunotherapy in patients with metastatic castration-resistant prostate cancer: a meta-analysis of data from 7 phase III studies and 3 phase II studies

**DOI:** 10.1186/s40164-022-00312-y

**Published:** 2022-09-26

**Authors:** Anqiang Zhang, Dali Tong

**Affiliations:** 1 State Key Laboratory of Trauma, Burns and Combined Injury, Wound Trauma Medical Center, Institute of Surgery Research, Daping Hospital, Army Medical University, Chongqing, China; 2 Department of Urology, Daping Hospital, Army Medical University, Chongqing, China

**Keywords:** Immunotherapies, ICBs, Vaccines, mCRPC, OS, PFS, ORR, TRAEs

## Abstract

**Background:**

Immunotherapies have emerged as potential treatments for metastatic castration-resistant prostate cancer (mCRPC). However, it is still unclear to identify the efficacy and safety of immunotherapy in large-scale samples. We performed a meta-analysis of 7 phase III randomized trials and 3 phase II trials comparing immunotherapy to placebo in mCRPC.

**Methods:**

Searching the PubMed, ClinicalTrials and Cochrane Library, completed III/IV phase trials were identified. Data extraction was conducted according to the PRISMA statement. The measured outcomes were OS, PFS, ORR and AE. Based on the results of phase III randomized trials, 3 II phase trials with results were identified.

**Results:**

A total of 4185 patients were available for evaluation of OS, and 3320 for PFS. Compared to placebo, immunotherapies were not able to improve OS (HR = 0.90; 95%CI 0.79–1.03; *p* = 0.13). However, immunotherapies, especially ICBs were able to decrease the risk of progression over placebo by 18% (HR = 0.82; 95%CI 0.68–1.00; *p* = 0.04). Significant ORR improvement was found in patients treated in ICBs (RR = 1.90; 95%CI 1.30–2.78; *p* < 0.001). Immunotherapies (OR = 1.01, 95% CI = 0.40–2.56; OR = 1.27, 95% CI = 0.72–2.25) were not associated with significant any grade TRAEs and 3–4 grade TRAEs. However, in subgroup analysis, ICBs (OR = 2.85, 95% CI = 2.27–3.57) and vaccines (OR = 0.78, 95% CI = 0.64–0.53) were associated with significant 3–4 grade TRAEs respectively. Moreover, ICBs alone induced positive PSA response [OR = 2.43(1.09–5.43), *P* = 0.03(I^2^ = 0%, *P* = 0.83)] and was effective in advanced PC even without classical therapies based on three phase II clinical trials about ICBs.

**Conclusions:**

Immunotherapies are not able to improve OS, but significantly improve PFS and ORR especially in ICBs treatment. Immunotherapies were not associated with significant TRAEs. However, in subgroup analysis, ICBs and vaccines were associated with significant 3–4 grade TRAEs.

**Supplementary Information:**

The online version contains supplementary material available at 10.1186/s40164-022-00312-y.

To the editor,

In USA, the proportion of prostate cancer (PC) diagnosed at a distant stage increased from 3.9% to 8.2% over the past decade. the incidence and mortality of PC are estimated to be 268,490 and 34,500 cases per years, respectively [1]. Although patients are effective to ADT in early-stage, most PCs finally develop to castration-resistant PC (CRPC) and metastatic CRPC (mCRPC) after a median-survival time. Although ADT and surgery, followed by chemotherapy and/or radiotherapy, remain the mainstay of mCRPC management, immunotherapy is rapidly being incorporated with other therapies to improve patient survival [2]. Considering the emerging roles of immunotherapy, we aimed to perform a meta-analysis of seven III-phase and three II-phase trials defining clinical outcome of two categories of immunotherapy: immune checkpoint blockers (ICBs) or vaccines in mCRPC patients.

This reviewed process led to the selection of 8 citations, which contains 7 III phase RCT trials considered for final meta-analysis (Fig. 1A and Additional file 1) [3–8]. A total of 4185 patients were available for evaluation of Overall survival (OS) while 3320 were evaluable for progression-free survival (PFS). The characteristics of each trial analyzed in this meta-analysis are shown in Table 1. Data for OS were available from all studies, with a total of 2506 patients treated with immunotherapy plus standard therapy (799 with ipilimumab and 1707 with vaccines), compared to 1679 patients treated with single standard therapy (602 with ipilimumab placebo and 1077 with vaccine placebo). Immunotherapy was not able to decrease the risk of death over placebo (HR = 0.90; 95%CI 0.79–1.03; *p* = 0.13) (Fig. 1B). Significant heterogeneity was found (Chi^2^ = 15.07, *p* = 0.02; I^2^ = 60.2%). Immunotherapies was not able to decrease the risk of death in patients with both ipilimumab (HR = 0.95; 95%CI 0.71–1.26; *p* = 0.71) and vaccine (HR = 0.88; 95%CI 0.73–1.05; *p* = 0.15) subgroups over placebo.

**Fig. 1:**
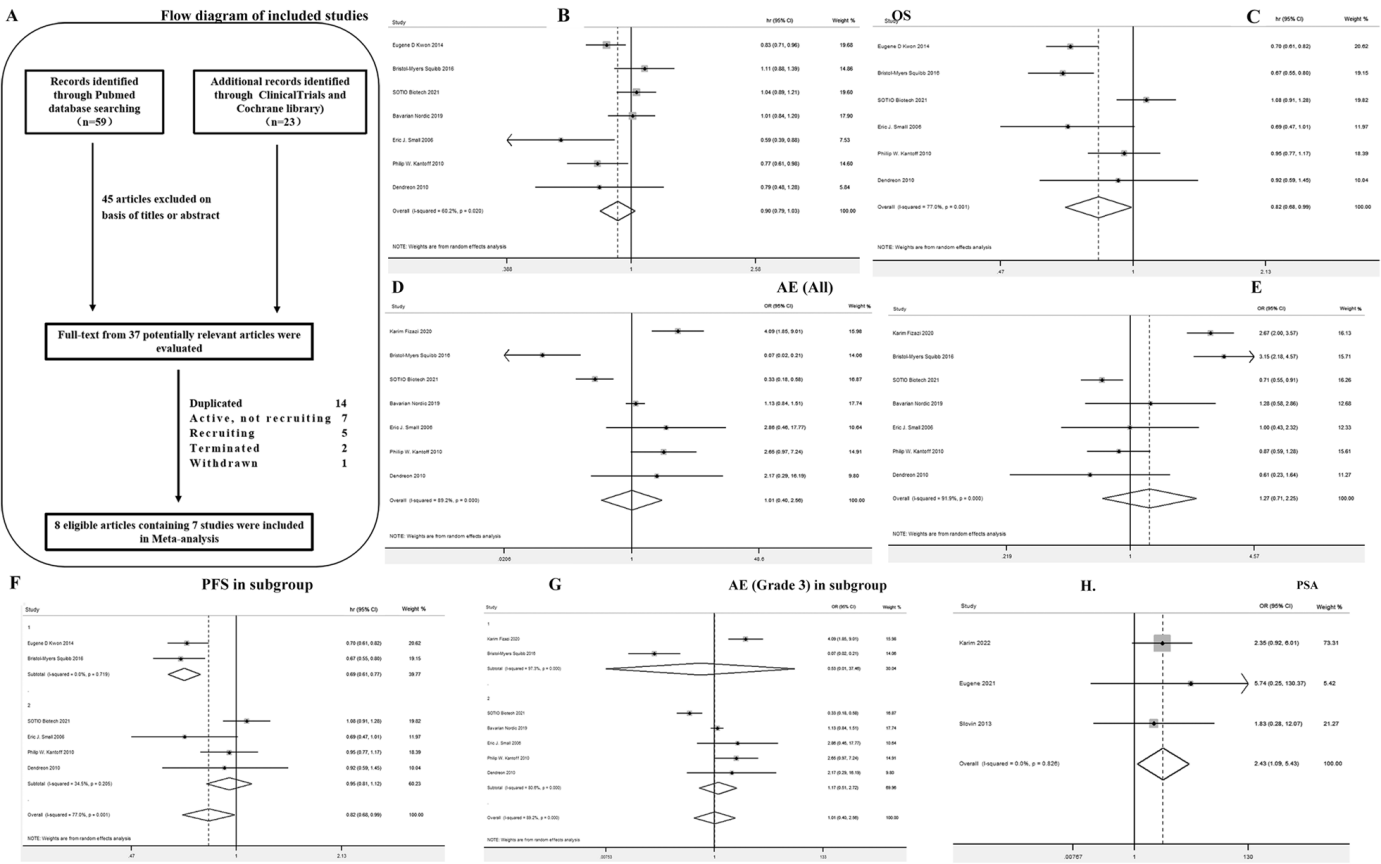
Flowchart and core results of the assessment of the studies identified in the meta-analysis. **A** Selection process for randomized controlled trials included in the meta-analysis. **B** Hazard ratio for overall survival in overall population treated with immunotherapy versus placebo shown as forest map. **C** Hazard ratio for progression free survival in overall population treated with immunotherapy versus placebo shown as forest map. **D**, **E** Odds ratio for safety estimation in any grade TRAEs (**D**) and 3–4 grade TRAEs (**E**) treated with immunotherapy versus placebo. **F** A subgroup analysis for PFS was performed in patients with ipilimumab and vaccine respectively. **G** A subgroup analysis on significant 3–4 grade TRAEs of ICBs and vaccines. **H** PSA response in ICBs group compared to ICBs plus first-line therapies

**Table 1:** Characteristics and summary of results for the studies of immunotherapy of metastatic castration-resistant prostate cancer included in the network meta-analysis

Trial	Experimental arm	Control arm	OS		PFS		ORR		Jadad’s score
			HR	95%CI	HR	95%CI	n/tot	n/tot	
NCT00861614 (CA184-043)	Ipilimumab	Placebo	0.83	0.71–0.96	0.7	0.61–0.82	211/393	133/396	3
NCT01057810 (CA184-095)	Ipilimumab	Placebo	1.11	0.88–1.39	0.67	0.55–0.80	202/399	43/199	3
NCT01133704 (D9902A)	Sipuleucel-T	APC-Placebo	0.79	0.48–1.28	0.92	0.59–1.45			3
NCT00065442 (D9902B)	Sipuleucel-T	APC-Placebo	0.78	0.61–0.98	0.95	0.77–1.17			3
NCT00005947 (D9901)	Sipuleucel-T	Placebo	0.59	0.39–0.88	0.69	0.47–1.01			3
NCT02111577 (SP005)	DCVAC/PCa With Standard of Care Chemotherapy	Placebo With Standard of Care Chemotherapy	1.042	0.90–1.21	1.08	0.91–1.28			3
NCT01322490 (BNIT-PRV-301)	PROSTVAC-V/F-TRICOM + GM-CSF Placebo	Placebo Control	1.01	0.84–1.20					3

Data for PFS were available from six studies except BNIT-PRV-301, with a total of 2074 patients treated with immunotherapy plus standard therapy (799 with ipilimumab and 1275 with vaccines), compared to 1246 patients treated with single standard therapy (602 with ipilimumab placebo and 644 with vaccine placebo). Immunotherapy was able to decrease the risk of progression over placebo by 18% (HR = 0.82; 95%CI 0.68–1.00; *p* = 0.04) (Fig. 1C). Significant heterogeneity was found (Chi^2^ = 21.72, *p* = 0.001; I^2^ = 77%). A subgroup analysis for PFS was performed in patients with ipilimumab and vaccine respectively. In ipilimumab subpopulation, immunotherapy was able to decrease the risk of progression over placebo by 31% (HR = 0.69; 95%CI 0.61–0.77; *p* = 0.000). No significant heterogeneity was found (Chi^2^ = 0.13, *p* = 0.72; I^2^ = 0%). In vaccine subpopulation, immunotherapy was not able to decrease the risk of progression over placebo (HR = 0.95; 95%CI 0.81–1.12; *p* = 0.546) (Fig. 1F).

Immunotherapy was able to increase the overall response rate (ORR) of ipilimumab over placebo (random effect, RR = 1.90; 95%CI 1.30–2.78; *p* < 0.001). Significant heterogeneity was found (Chi^2^ = 5.45, *p* = 0.02; I2 = 82%). Immunotherapies were not significantly associated with any grade treatment-related adverse events (TRAEs) (OR = 1.01, 95% CI = 0.40–2.56) (Fig. 1D) and 3–4 grade TRAEs (OR = 1.27, 95% CI = 0.72–2.25) (Fig. 1E). However, in subgroup analysis, ICBs (OR = 2.85, 95% CI = 2.27–3.57) and vaccines (OR = 0.78, 95% CI = 0.64–0.53) were associated with significant 3–4 grade TRAEs (Fig. 1G).

A series of III phase trails is ongoing (Additional file 2: Table S1). At present, most PC immunotherapy studies are still in phase II trials. Three trials were included about Ipilimumab and Nivolumab after screening (Additional file 3). We found that obvious PSA response in ICBs group compared to ICBs plus first-line therapies (OR = 2.43(1.09–5.43), *P* = 0.03(I^2^ = 0%, *P* = 0.83)) (Fig. 1H), without significant differentiation about ORR [OR = 1.66(0.56–4.88), *P* = 0.36(I^2^ = 0%, *P* = 0.58)]. These results were not only in line with our analysis on phase III trials that immunotherapies impede PC progression, but also indicated that ICBs alone were effective in advanced PC even without classical therapies. These meta-analysis findings have not been reported elsewhere before.

The meta-analysis found that PC patients can benefit from immunotherapies. Our analysis suggested that immunotherapies are not able to improve OS, but significantly improve PFS and ORR especially in ICBs treatment. Immunotherapies were not associated with significant TRAEs. However, in subgroup analysis, ICBs and vaccines were associated with significant 3–4 grade TRAEs. In order to elaborate in-depth analysis, we compared three types of therapy respectively for sub-group meta-analysis based on clinical endpoints OS and PFS. Our data indicated that Sipuleucel-T is effective to improve OS, while ipilimumab is effective to improve PFS (Additional file 4). Collectively, in this double-blind, placebo-controlled, multicenter phase 3 trial, 512 patients were randomly assigned to receive either sipuleucel-T (341 patients) or placebo (171 patients) administration in a 2:1 ratio. The primary end point was overall survival (OS). Sipuleucel-T group induced a relative reduction of 22% in the risk of death as compared with the placebo group (HR = 0.78; 95% CI 0.61 to 0.98; *P* = 0.03), which represented a 4.1-month improvement in median survival (25.8 months/sipuleucel-T vs. 21.7 months/placebo). The 36-month survival probability was 31.7% administered by sipuleucel-T versus 23.0% administered by placebo. The adjusted HR for death was 0.78 (95% CI 0.61 to 0.98), representing a relative reduction in the risk of death of 22% (*P* = 0.03) for the sipuleucel-T group compared to placebo group, which were also obtained based on the unadjusted, stratified model and the log-rank test (HR = 0.77; 95% CI 0.61 to 0.97; *P* = 0.02). PSA reductions of at least 50% on follow-up were observed in 2.6% patients in the sipuleucel-T group compared to 1.3% patients in the placebo group. Both immune responses to the immunizing antigen and adverse events were more frequently observed in patients received sipuleucel-T. PFS was 14.6 weeks (3.7 months) in the sipuleucel-T group and 14.4 weeks (3.6 months) in the placebo group (HR = 0.95; 95% CI 0.77 to 1.17; *P* = 0.63). Sipuleucel-T prolonged OS for mCRPC patients, though no clinical benefits on PFS was observed [6]. Our in-depth meta-analysis further supported these results (Additional file 4).

Current standard of care in mCRPC with MSI-high and/or high TMB recommends pembrolizumab after all available and feasible treatment options. In fact, the updated treatment option for tumor have focused on the same pathological molecular phenotype, not different types of tumors, which is regarded as tumor agnostic therapies. Tumor agnostic therapies is a diagnosis and treatment concept for different tumors and the same pathological molecular phenotype. Tumor treatment is no longer differentiated by site, but is administered according to the same gene abnormality. This innovative diagnosis and treatment method brings new treatment options for cancer patients. Since 2017, the FDA has approved six drugs with histology-agnostic indications: pembrolizumab (mismatch-repair deficiency (dMMR)/high microsatellite instability (MSI-H) phenotype/high tumor mutational burden (TMB-H) phenotype), dostarlimab, larotrectinib and entrectinib, and the combination of dabrafenib plus trametinib. The efficacy of pembrolizumab in dMMR/MSI-H mCRPC has been confirmed [9].

Particularly, pembrolizumab is first approved to use for advanced solid tumors with the same genetic abnormalities (MSI-H or dMMR). Based on a combined analysis of five clinical trials (KN-164, KN-012, KN-028, KN-158, and KN-016), the FDA granted the approval of pembrolizumab as the first tissue-agnostic drug for solid tumors in 2017 (https://www.fda.gov/drugs/resources-information-approved-drugs/fda-grants-accelerated-approval-pembrolizumab-first-tissuesite-agnostic-indication). KN-016 trial reported that pembrolizumab showed an improvement in terms of the objective response rate (ORR) and median PFS for dMMR colorectal cancer (CRC) and non-CRC patients compared to pMMR CRC patients in a cohort of 41 patients with treatment-refractory metastatic carcinomas. Further study was expanded to investigate the effect of pembrolizumab in 86 patients with 12 different metastatic dMMR tumor types. The results showed an ORR of 53% (95% CI 42–64%), and complete responses (CRs) were achieved in 21% of patients. Among 233 enrolled patients treated with pembrolizumab and affected by 27 tumor types, the ORR was 34.3% (95% CI 28.3–40.8%), and the mPFS was 4.1 months (95% CI 2.4–4.9 months) (Additional file 5). Furthermore, only a small number of mCRPC patients were enrolled in the clinical trials that led to the approval of the above-mentioned drugs. The efficacy of pembrolizumab in dMMR/MSI-H mCRPC has been confirmed. A multicenter retrospective study describing the clinical features of 27 dMMR/MSI-H mCRPC patients and their responses to PD-1 blockade reported PSA response for 15 out of the 17 patients who received pembrolizumab, which showed PSA50 occurred in eight (53%) patients after six-month follow-up for estimated PFS at 64.1% (95% CI 33.7–83.4%) [10]. Based on the data from first case series reporting the clinical activity of pembrolizumab for dMMR/MSI-H mCRPC, 9 patients were treated with pembrolizumab, of which 4 patients achieved PSA50 after treatment process, including three patients with a PSA decline greater than 99% [11].

As prospectively planned retrospective analysis, KN-158 trial investigated the activity of pembrolizumab in patients with pre-treated unresectable or metastatic TMB-H solid tumors (TMB ≥ 10 mut/Mb). The ORR was 29% (95% CI 21–39%) in the TMB-H group, with 4% CR and 25% PR compared to in the non-TMB-H group was 6% (95% CI 5–8%) after a median follow-up of 37.1 months (Additional file 5). In 2020, the FDA approved pembrolizumab for the treatment of adult and pediatric patients with unresectable or metastatic TMB-H (≥ 10 mut/Mb) solid tumors that progressed on prior treatments and with no alternative therapeutic options. A comparative study to assess the therapy outcomes of mCRPC patients receiving immune checkpoint inhibitors (ICIs, 45 patients, 75.6% received pembrolizumab, 20% nivolumab, and 4.4% atezolizumab) compared to taxane chemotherapy (696 patients) showed a worse median time to next therapy among patients with TMB < 10 mt/Mb receiving ICIs than for those receiving taxanes (2.4 vs. 4.1 months; HR = 2.65; 95% CI 1.78–3.95; *p* < 0.001). In contrast, for patients with TMB ≥ 10 mt/Mb, compared with taxanes, ICIs showed more favorable outcomes (8.0 vs. 2.4 months; HR = 0.37; 95% CI 0.15–0.87; *p* = 0.02) and overall survival (19.9 vs. 4.2 months; HR = 0.23; 95% CI 0.10–0.57; *p* = 0.001) [12].

## Supplementary Information

**Additional file 1:** The materials and methods section of meta-analysis including the descriptions of study outcome, inclusion and exclusion criteria, search strategy, study selection, data extraction and quality assessment and statistical analysis.

**Additional file 2: Table S1.** Ongoing III phase randomized trials with immunotherapy in mCRPC.

**Additional file 3:** Three phase II clinical trials were included about Ipilimumab and Nivolumab after screening. In detail, most prostate cancer immunotherapy studies are still in phase II clinical trials, and the number of clinical trials above phase III is very limited. We further screened 86 phase II clinical trials about ICBs, and three trials about Ipilimumab and Nivolumab were included based on maintenance therapy.

**Additional file 4:** Three types of therapy based on ipilimumab, sipuleucel-T and two vaccines respectively for subgroup meta-analysis based on clinical endpoints OS and PFS were performed. In term of OS, immunotherapy based on sipuleucel-T subgroup were able to decrease the risk of death in patients (HR = 0.73; 95%CI, 0.61–0.88; p = 0.001), indicating that sipuleucel-T is effective to improve OS. In term of PFS, in ipilimumab subpopulation, immunotherapy was able to decrease the risk of progression over placebo by 31% (HR = 0.69; 95%CI, 0.61–0.77; p = 0.000), which indicates that ipilimumab is effective to improve PFS.

**Additional file 5:** The trials about pembrolizumab approved to use for advanced solid tumors with the multiple genetic abnormalities (MSI-H or dMMR or TMB-H) were reviewed based on objective response rate (ORR), PFS and complete responses (CRs).

## Data Availability

The datasets used and/or analyzed during the current study are available from the corresponding author on reasonable request.
