# Supplementary material for: Immunotherapy in patients with metastatic castration-resistant prostate cancer: a meta-analysis of data from 7 phase III studies and 3 phase II studies
Source: Exp Hematol Oncol. 2022 Sep 26;11:63. doi: 10.1186/s40164-022-00312-y (PMC9511714; doi:10.1186/s40164-022-00312-y)
Supplement: Supplementary file 1 — Additional file 1: The materials and methods section of meta-analysis including the descriptions of study outcome, inclusion and exclusion criteria, search strategy, study selection, data extraction and quality assessment and statistical analysis. [file 40164_2022_312_MOESM1_ESM.docx]

**Materials and methods**

*Study Outcomes*

Immunotherapy was regarded as the experimental arm, while placebo as the control arm in the analysis. The OS and the PFS assessed in view of the hazard ratios (HR) and 95% confidence intervals (CIs) for the experimental arm over the control arm. The overall response rate (ORR) was assessed in view of the risk ratio (RR) and 95% CIs for the experimental arm over the control arm.

*Inclusion and exclusion criteria*

The inclusion criteria were as follows: III/IV phase randomized trials reporting data in mCRPC patients except conference abstracts. The exclusion criteria were as follows: overlapping publications, terminal trials without relevant outcome data, and ongoing trials with data not yet reported.

*Search strategy*

PubMed, ClinicalTrials and Cochrane Library database were reviewed with limit to phase III or phase IV clinical trials. The key terms were “prostate cancer”, “immunotherapy”, “ipilimumab”, “nivolumab”, “atezolizumab” and “pembrolizumab”.

*Study selection*

The electronic search identified 82 citations, of which 22 articles were used for further assessment, and 14 citations were excluded without meeting the inclusion criteria. This reviewed process led to the selection of 8 citations, which contains 7 III phase RCT trials considered for final analysis based on their adequate quality and relevance for inclusion in the meta-analysis. All studies enrolled mCRPC patients based on disease progression followed by hormonal therapy with or without radiotherapy and chemotherapy. All studies have also updated 3-years follow up results. Two studies (CA184-043 and CA184-095) compared the combination of ipilimumab (CTLA-4 inhibitor) plus standard therapy to single standard therapy; five studies (D9902A, D9902B, D9901, SP005 and BNIT-PRV-301) compared the combination of various types of vaccines plus standard therapy to single standard therapy, of which D9902A, D9902B and D9901 were associated with sipuleucel-T, SP005 associated with DCVAC/PCa and BNIT-PRV-301 associated with PROSTVAC.

*Data extraction*

Data were independently extracted from the selected studies by two authors (AZ, DT) into a standard Microsoft^®^ Excel^®^ spreadsheet. Any disagreement was resolved by consensus between these two authors. The following data were extracted from the included studies: name of first author, publication year, clinical trial phase, patient number, each number of two arms, treatment type, and HRs with 95% CI for PFS, HRs with 95% CI for OS and patient number with tumor response in each arm. The meta-analyses were performed in overall population and subgroup analyses were performed in patients treated in checkpoint inhibitors and vaccines respectively.

*Quality assessment*

Quality assessment was based on the Jadad 5-item scale with the final score ranged from 0 to 5. This meta-analysis included III phase unblinded and randomized clinical trials. The Jadad’s score was evaluated as 3 for all the studies.

*Statistical analysis*

The HRs with the 95% CIs for PFS and OS were extracted based on each study for immunotherapy arm over control arm. If heterogeneity between studies was found to be significant, the random-effects model was used. Otherwise, the fixed-effects model was used. Results were illustrated using forest plots. A two-tailed P<0.05 was considered statistically significant. The meta-analysis was performed using the RevMan software (v.5.3).
