# Supplementary material for: Immunotherapy in patients with metastatic castration-resistant prostate cancer: a meta-analysis of data from 7 phase III studies and 3 phase II studies
Source: Exp Hematol Oncol. 2022 Sep 26;11:63. doi: 10.1186/s40164-022-00312-y (PMC9511714; doi:10.1186/s40164-022-00312-y)
Supplement: Supplementary file 2 — Additional file 2: Table S1. Ongoing III phase randomized trials with immunotherapy in mCRPC. [file 40164_2022_312_MOESM2_ESM.pdf]

Table S1. Ongoing III phase randomized trials with immunotherapy in mCRPC

Trial	Experimental arm	Control arm	Primary endpoint	Phase	State	Last updated
NCT04100018	Nivolumab + Docetaxel + Prednisone	Placebo + Docetaxel + Prednisone	OS, rPFS	III	Recruiting	January 11, 2022
NCT04446117	Cabozantinib + Atezolizumab	Abiraterone+ Prednisone /Enzalutamide	PFS, OS	III	Recruiting	December 2, 2021
NCT03016312	Atezolizumab + Enzalutamide	Enzalutamide	OS	III	Active, not recruiting	January 12, 2022
NCT03834493	Pembrolizumab + Enzalutamide	Placebo + Enzalutamide	OS, rPFS	III	Recruiting	February 17, 2022
NCT04907227	Pembrolizumab+ Docetaxel	Placebo+Docetaxel	OS, rPFS	III	Recruiting	July 7, 2021
NCT04934722	Pembrolizumab + Enzalutamide + ADT	Placebo + Enzalutamide + ADT	OS, rPFS	III	Recruiting	September 8, 2021
NCT03834506	Pembrolizumab+ Docetaxel	Placebo+Docetaxel	OS, rPFS	III	Active, not recruiting	June 9, 2021
NCT03834519	Pembrolizumab + Olaparib	Abiraterone + Prednisone or Enzalutamide	OS, rPFS	III	Active, not recruiting	September 16, 2021
