# Supplementary material for: Immunotherapy in patients with metastatic castration-resistant prostate cancer: a meta-analysis of data from 7 phase III studies and 3 phase II studies
Source: Exp Hematol Oncol. 2022 Sep 26;11:63. doi: 10.1186/s40164-022-00312-y (PMC9511714; doi:10.1186/s40164-022-00312-y)
Supplement: Supplementary file 3 — Additional file 3: Three phase II clinical trials were included about Ipilimumab and Nivolumab after screening. In detail, most prostate cancer immunotherapy studies are still in phase II clinical trials, and the number of clinical trials above phase III is very limited. We further screened 86 phase II clinical trials about ICBs, and three trials about Ipilimumab and Nivolumab were included based on maintenance therapy. [file 40164_2022_312_MOESM3_ESM.docx]

At present, most prostate cancer immunotherapy studies are still in phase II clinical trials, and the number of clinical trials above phase III is very limited, resulting in the inability to develop and analyze large-scale clinical data. We further screened 86 phase II clinical trials about ICBs, and three trials about Ipilimumab and Nivolumab were included based on maintenance therapy. We found that obvious PSA response in ICBs alone group compared to ICBs plus first-line therapies (OR=2.43(1.09-5.43), P=0.03(I^2^ = 0%, P=0.83)). However, there is no significant differentiation about overall response between the two groups (OR=1.66(0.56-4.88), P=0.36(I^2^ = 0%, P=0.58))[[1-3](#_ENREF_9)]. These results were not only in line with our analysis on phase III trials that immunotherapies can impede PC progression, but also indicated that ICBs alone were effective in advanced PC even without classical therapies. This benefit of immune cell activation will be reached based on ICBs.

[1] Shenderov E, Boudadi K, Fu W, Wang H, Sullivan R, Jordan A, et al. Nivolumab plus ipilimumab, with or without enzalutamide, in AR-V7-expressing metastatic castration-resistant prostate cancer: A phase-2 nonrandomized clinical trial. Prostate. 2021;81:326-38.

[2] Slovin SF, Higano CS, Hamid O, Tejwani S, Harzstark A, Alumkal JJ, et al. Ipilimumab alone or in combination with radiotherapy in metastatic castration-resistant prostate cancer: results from an open-label, multicenter phase I/II study. Ann Oncol. 2013;24:1813-21.

[3] Fizazi K, Gonzalez Mella P, Castellano D, Minatta JN, Rezazadeh Kalebasty A, Shaffer D, et al. Nivolumab plus docetaxel in patients with chemotherapy-naive metastatic castration-resistant prostate cancer: results from the phase II CheckMate 9KD trial. Eur J Cancer. 2022;160:61-71.
