# Supplementary material for: Immunotherapy in patients with metastatic castration-resistant prostate cancer: a meta-analysis of data from 7 phase III studies and 3 phase II studies
Source: Exp Hematol Oncol. 2022 Sep 26;11:63. doi: 10.1186/s40164-022-00312-y (PMC9511714; doi:10.1186/s40164-022-00312-y)
Supplement: Supplementary file 4 — Additional file 4: Three types of therapy based on ipilimumab, sipuleucel-T and two vaccines respectively for subgroup meta-analysis based on clinical endpoints OS and PFS were performed. In term of OS, immunotherapy based on sipuleucel-T subgroup were able to decrease the risk of death in patients (HR = 0.73; 95%CI, 0.61–0.88; p = 0.001), indicating that sipuleucel-T is effective to improve OS. In term of PFS, in ipilimumab subpopulation, immunotherapy was able to decrease the risk of progression over placebo by 31% (HR = 0.69; 95%CI, 0.61–0.77; p = 0.000), which indicates that ipilimumab is effective to improve PFS. [file 40164_2022_312_MOESM4_ESM.docx]

In order to elaborate in-depth analysis, we compared three types of therapy respectively for sub-group meta-analysis based on clinical endpoints OS and PFS. In term of OS, immunotherapies were not able to decrease the risk of death in patients with both ipilimumab (HR = 0.95; 95%CI, 0.71–1.26; p=0.71) and two vaccine (HR = 1.03; 95%CI, 0.92–1.16; p=0.64) subgroups over placebo. However, immunotherapy based on sipuleucel-T subgroup were able to decrease the risk of death in patients (HR = 0.73; 95%CI, 0.61–0.88; p=0.001), which indicates that sipuleucel-T is effective to improve OS. In term of PFS, in ipilimumab subpopulation, immunotherapy was able to decrease the risk of progression over placebo by 31% (HR = 0.69; 95%CI, 0.61–0.77; p=0.000), which indicates that ipilimumab is effective to improve PFS. However, immunotherapies were not able to decrease the risk of death in patients with both vaccine (HR = 1.08; 95%CI, 0.91–1.28; p=0.392) and sipuleucel-T (HR = 0.89; 95%CI, 0.74–1.06; p=0.178) subgroups over placebo.
