# Supplementary material for: Immunotherapy in patients with metastatic castration-resistant prostate cancer: a meta-analysis of data from 7 phase III studies and 3 phase II studies
Source: Exp Hematol Oncol. 2022 Sep 26;11:63. doi: 10.1186/s40164-022-00312-y (PMC9511714; doi:10.1186/s40164-022-00312-y)
Supplement: Supplementary file 5 — Additional file 5: The trials about pembrolizumab approved to use for advanced solid tumors with the multiple genetic abnormalities (MSI-H or dMMR or TMB-H) were reviewed based on objective response rate (ORR), PFS and complete responses (CRs). [file 40164_2022_312_MOESM5_ESM.docx]

KN-016 trial reported that pembrolizumab showed an improvement in terms of the objective response rate (ORR) and median PFS for dMMR colorectal cancer (CRC) and non-CRC patients compared to pMMR CRC patients in a cohort of 41 patients with treatment-refractory metastatic carcinomas[[1](#_ENREF_1)]. Further study was expanded to investigate the effect of pembrolizumab in 86 patients with 12 different metastatic dMMR tumor types. The results showed an ORR of 53% (95% CI, 42–64%), and complete responses (CRs) were achieved in 21% of patients[[2](#_ENREF_2)]. Among 233 enrolled patients treated with pembrolizumab and affected by 27 tumor types, the ORR was 34.3% (95% CI, 28.3–40.8%), and the mPFS was 4.1 months (95% CI, 2.4–4.9 months)[[3](#_ENREF_3)].

As prospectively planned retrospective analysis, KN-158 trial investigated the activity of pembrolizumab in patients with pre-treated unresectable or metastatic TMB-H solid tumors (TMB ≥ 10 mut/Mb). The ORR was 29% (95% CI, 21%–39%) in the TMB-H group, with 4% CR and 25% PR compared to in the non-TMB-H group was 6% (95% CI, 5–8%) after a median follow-up of 37.1 months[[4](#_ENREF_4)].

1. Le DT, Uram JN, Wang H, Bartlett BR, Kemberling H, Eyring AD, Skora AD, Luber BS, Azad NS, Laheru D *et al*: **PD-1 Blockade in Tumors with Mismatch-Repair Deficiency**. *N Engl J Med* 2015, **372**(26):2509-2520.

2. Le DT, Durham JN, Smith KN, Wang H, Bartlett BR, Aulakh LK, Lu S, Kemberling H, Wilt C, Luber BS *et al*: **Mismatch repair deficiency predicts response of solid tumors to PD-1 blockade**. *Science* 2017, **357**(6349):409-413.

3. Marabelle A, Le DT, Ascierto PA, Di Giacomo AM, De Jesus-Acosta A, Delord JP, Geva R, Gottfried M, Penel N, Hansen AR *et al*: **Efficacy of Pembrolizumab in Patients With Noncolorectal High Microsatellite Instability/Mismatch Repair-Deficient Cancer: Results From the Phase II KEYNOTE-158 Study**. *J Clin Oncol* 2020, **38**(1):1-10.

4. Marabelle A, Fakih M, Lopez J, Shah M, Shapira-Frommer R, Nakagawa K, Chung HC, Kindler HL, Lopez-Martin JA, Miller WH, Jr. *et al*: **Association of tumour mutational burden with outcomes in patients with advanced solid tumours treated with pembrolizumab: prospective biomarker analysis of the multicohort, open-label, phase 2 KEYNOTE-158 study**. *Lancet Oncol* 2020, **21**(10):1353-1365.
